# A quantitative exploration of the sociocultural context of teenage pregnancy in Sri Lanka

**DOI:** 10.1186/s12884-014-0394-y

**Published:** 2014-12-05

**Authors:** Neelamani Rajapaksa-Hewageegana, Sarah Maria Salway, Hilary Piercy, Sarath Samarage

**Affiliations:** Health Education Bureau, 2 Kynsey Road, Colombo, 08 Sri Lanka; Sarah Salway, School of Health & Related Research, University of Sheffield, 30 Regent Street, Sheffield, S14DA UK; Hilary Piercy, Centre for Health & Social Care Research, Sheffield Hallam University, 34 Collegiate Crescent, Sheffield, S102BP UK; Sarath Samarage, WHO Country Office, 226 Bauddhaloka Mawatha, Colombo, 7 Sri Lanka

## Abstract

**Background:**

In common with other countries, teenage pregnancy is attracting policy attention in Sri Lanka because of the risks it poses to maternal and infant health and social and economic well-being. This study aimed to increase understanding of the context of teenage pregnancy, by (1) describing the socio-economic and demographic characteristics of pregnant teenagers and their partners; (2) exploring whether teenage pregnancies are planned and how they are received; and (3) exploring factors associated with unplanned teenage pregnancy.

**Methods:**

A population health-register based sample survey was conducted in Badulla District, Sri Lanka. Interviewer-administered questionnaires were administered to two samples: 450 pregnant women aged less than 20 years; and 150 male partners of pregnant women aged less than 20 years. Bivariate statistics described the characteristics and context of teenage pregnancy. Multivariate logistic regression explored correlates of unplanned pregnancy.

**Results:**

Over 60% of pregnant teenagers and male partners indicated that the current pregnancy was planned; while 79% of pregnant teenagers and 85% of male partners welcomed the pregnancy. Most pregnant teenagers were living within stable and supportive family environments, with 94% reporting that they felt ‘very well supported’. Nevertheless, a sub-group of pregnant teenagers appeared to be vulnerable, reporting unplanned and unhappy pregnancy; factors that were also associated with first intercourse being reported as not wanted. Levels of reproductive and contraceptive knowledge were poor among both pregnant teenagers and male partners. Just 46% of teenagers and 64% of male partners knew that pregnancy was possible at first intercourse. Mothers appear to be an important source of information and support for young women, with peers being reported far less often.

**Conclusions:**

Intervention to reduce teenage pregnancy must recognise the normative nature of early childbearing for the majority of girls who currently conceive and their families. Avoiding such pregnancies will require a fundamental shift in life chances such that delaying pregnancy offers significant socioeconomic advantages. Meanwhile, improved provision of contraceptive information and services is needed to support the delay of second pregnancies for young mothers. In addition, strategies to identify and protect those girls who are vulnerable to unwanted sexual activity are needed.

## Background

In common with many parts of the world, teenage pregnancy is attracting increasing policy attention in Sri Lanka because of the risks it poses to maternal and infant health as well as to social and economic well-being [1]. Despite low levels compared to neighbouring countries and evidence of a decline in recent years, important differentials persist between regions, socioeconomic and ethnic groups [2]. Furthermore, there are concerns that pregnancy may have devastating consequences for teenage girls when it does occur, particularly if it happens outside of marriage [3,4]. Goonewardne and Waduge reported that 54% of pregnancies among 13–16 year olds and 23% among 17–19 year olds in their hospital-based study were unplanned [3], while an earlier study reported an overall unplanned pregnancy rate of 62% in their sample of 113 teenage mothers [5]. A more recent, population-level study reports that around 20% of teenage pregnancies were unplanned [6]. Further, the limited data available suggest that a substantial proportion of those seeking termination of pregnancy (which is illegal in Sri Lanka) are adolescents [7,8]. Evidence also indicates poorer birth outcomes across a range of indicators in those studies that have compared teenage to older mothers [3].

However, to-date there has been little exploration of the patterns, determinants or context of teen pregnancy in Sri Lanka. A recent systematic review of factors associated with teenage pregnancy in South Asia, identified just two Sri Lankan studies, both of them hospital based [9]. These studies reported low education and low socioeconomic status to be associated with teenage pregnancy [5,10]. Other research indicates that lack of contraceptive knowledge and fear of side effects contribute to low levels of contraceptive usage in general [5,] [Linganathan K: *Factors associated with teenage pregnancy,* unpublished], whilst unmarried individuals face additional difficulties in accessing contraceptive services [11]. A more recent study in three districts has confirmed that low education is associated with teenage pregnancy [12,13]. These authors also suggested that aspects of an unsupportive family environment may characterise pregnant teenagers, while other research has suggested that the majority of pregnant teenagers have parental support [3]. Divergent findings and evidence of significant geographical variation, as well as unexamined factors, underscore the need for further exploration of the sociocultural context of teenage pregnancies in Sri Lanka.

As well as limited local understanding, there are significant challenges to extrapolating evidence from other settings – such as the USA, UK or even other South and Southeast Asian countries - given the important differences in socio-cultural milieu, marriage and childbearing practices [14,15]. There is therefore a growing demand for locally-relevant evidence on this topic as evidenced in existing and forthcoming policy documents including the National Youth Policy [16-18].

The present study aimed to increase understanding of the context within which teen pregnancy occurs in Sri Lanka; a vital first step to inform policy and practice development in this area. A population-based study was undertaken in one district in Sri Lanka with the objectives to: (i) describe the socio-economic and demographic characteristics of pregnant teenagers and their partners; (ii) explore whether teenage pregnancies are planned and how they are received; and (iii) explore factors that may increase the likelihood of unplanned teenage pregnancy. The paper importantly extends earlier work by: drawing on a population-register based sample in a region so far not explored; examining a wide range of potential predictive factors including religio-ethnic differentials; and including the male partners of pregnant teenagers.

## Method

### Study context

The study was conducted in Badulla in the central hill country. The district has around 860,000 residents and socioeconomic characteristics comparable with those in other parts of the country [19]. The teen pregnancy rate is 7.2%, a little above the national average, with higher rates among those living on the tea plantations and the indigenous Veddha population [Regional Director of Health Services Badulla: unpublished]. The area is divided into fifteen medical officer of health (MOH) areas for provision of community health services. Maternity services, including antenatal care, are delivered by government employed public health midwives each serving a population of approximately 3,000.

### Recruitment of study participants

The study population comprised all girls aged 19 years or younger registered with public maternity services in the two year period July 2007 - June 2009. Given the very high coverage rate of antenatal care – estimated to be 99% [19]- the midwives’ registers provided a very robust sampling frame from which to recruit pregnant teenagers (regardless of their marital status). Based on a desire to estimate the proportion of pregnant teenagers who were married (conservatively estimated to be 50%) with an accuracy of +/−5% and an anticipated non-response rate of 15% and using standard methods for sample size calculation [20], a target sample size of 450 was set.

The total target sample of 450 pregnant adolescents was stratified across MOH areas in proportion to the adolescent pregnancy rate in each area in the year prior to data collection. In those ten MOH areas that included plantation populations, the target sample size was further stratified across plantation and non-plantation areas in proportion to these population sizes (with one fifth of the samples being drawn from plantation areas). This method ensured that the sample included proportionate representation of the urban, rural and plantation population.

For the non-plantation sample, all the midwives’ names (each of whom covers a distinct area) were written down in alphabetical order by each MOH area. Next, these listed names were randomly reorganised within each MOH area. Upcoming clinic schedules for each midwife were collected and listed. Interviewers then visited the next available clinic for the first randomly selected midwife and recruited respondents to the study as detailed below. Interviewers proceeded to visit midwives in their clinics in the randomly assigned order until the target sample size for that MOH area had been reached. In those ten MOH areas that included plantation populations, the plantations were listed in alphabetical order and then randomly reorganised. As before, clinic timings were ascertained and the next available clinic was visited for each plantation in random order until the target sample size had been reached. In total, 179 non-plantation midwives out of a total of 249 were visited and 21 plantations out of a total of 60 were visited, giving a well-distributed sample.

When data collectors visited the clinics they adopted the following procedure. All pregnant teenagers who were due to attend the clinic that day were identified from the midwife’s register of pregnant women. Every second pregnant teenager was selected and, on arrival at the clinic, these individuals were invited to participate in the study by the clinic midwife. If the teenager expressed verbal consent to participate, one of the three data collectors (either a public health nursing sister or a medical assistant) then explained the study in more detail, gave the respondent time to consider the study, answered any questions and took written consent to participation before administering the questionnaire. If the pregnant teenager was under 18 years, permission was also sought from her parents, guardians or partner who had accompanied her to the clinic. Participant recruitment for the study continued until the target number of pregnant teenagers was achieved for each of the MOH areas; ranging from 63 respondents in Welimada MOH area to 12 in Soranathota area. Each clinic visit yielded one (in 92 visits), two (in 42 visits) or more completed questionnaires, up to a maximum of eight (in just four visits).

For partners of pregnant teenagers, a pragmatic sample size of 150 was determined on the basis of available resources. All partners of pregnant girls aged 19 years or under in the district of study were eligible and recruitment began in July 2008. As with pregnant teenagers, the required sample size was stratified across the MOH areas and the plantation populations. Since partners did not always attend clinics, unlike the pregnant adolescents themselves, a modified approach was used to locate partner respondents. In each MOH area, a randomly selected group of midwives were asked to identify pregnant teenagers in their field registers and to extend an initial invitation to male partners to participate in the study. If a positive response was received, the partner’s details were passed to a male Public Health Inspector employed in the district who then made contact and arranged a mutually convenient time and location to explain the study, take consent and administer the questionnaire. Again, recruitment continued in each area until the target sample size was achieved, ensuring representation across the district.

### Data collection

In the absence of any theoretically-informed work in the Sri Lankan context, an exploratory approach was taken based on a combination of conceptual insights and empirical findings from other contexts [21-24]. This earlier work raised a host of potential pathways of influence on adolescent pregnancy and suggested the importance of exploring: socio-economic circumstances; connectedness to family and family setting; school influences and experiences; partner characteristics; knowledge levels; peer and adolescent norms; and community norms. Structured questionnaires were developed for one-time administration to both pregnant teenagers (aged less than 20 years) and the partners of pregnant teenagers. The content of the questionnaires was largely similar across the two (though the partner questionnaire was somewhat shorter) and covered a range of topics including: socio-economic background; social circumstances; reproductive and contraceptive knowledge and practice; and planning and reaction to the pregnancy (ascertained via a series of questions). The questionnaires were initially prepared in English with input from academics and health practitioners, and then translated to Tamil and Sinhala, the local languages, and back-translated to English by a third party to ensure accuracy. Piloting was also undertaken before the questionnaires were finalised and administered by experienced interviewers (two female and one male) to both the pregnant teenagers and the partners of pregnant teenagers.

### Data analysis

Data underwent consistency, logical and range checks prior to analysis in SPSSX. Descriptive statistics were next performed and all variables were explored carefully to identify any inconsistencies, ensure credibility of the data, and to determine appropriate categorisations for open ended and categorical questions. Reproductive health knowledge was assessed through a range of questions the answers to which were recorded as ‘correct’ or ‘incorrect’. Background information is presented on the total sample. Subsequent analysis of factors describing the context within which first pregnancy occurred was confined to the 409 girls who were pregnant for the first time since answers to these questions would require a long period of recall for the 41 respondents who were pregnant for the second or third time, and circumstances of second and third pregnancies are likely to be very different. Cross tabulation and binary logistic regression analysis were undertaken to explore differentials between those who reported their pregnancy to be planned and those who reported it was not planned. Variables explored in the regression analysis were grouped according to potentially explanatory pathways: socio-economic circumstances; family setting; experiences of school; reproductive health knowledge; partner characteristics and community norms (proxy ethnicity).

Ethical approval for the study was received from the Sri Lanka Medical Association and approval to undertake the study from the line Ministry of Health, Provincial Ministry of Health and Provincial Director of Education.

## Results

### Socio-economic and demographic characteristics of pregnant teenagers and their partners

Table 1 presents descriptive socio-demographic characteristics of the pregnant teenagers. Almost one third (29%, n = 130) of the pregnant teenagers were under 18 years, with four being 14 years of age. Moors (12%) were over-represented in the sample as compared to their population proportion in the region (around 5%), indicating a higher teenage pregnancy rate in this group than among the majority Sinhalese and the Tamils who made up 76% and 12% of the sample respectively (compared to 72% and 22% in the local population) [19]. As expected for Sri Lanka, the vast majority (90%) demonstrated good literacy; 57% had received schooling to at least grade 10 and 52% had achieved academic qualifications. Just six (1.5%) had received no formal education, whilst 7% (n = 30) could not read at all. The majority (80%) reported positive schooling experiences. 85% reported a parental financial state of at least ‘break even’ at the time they met their current partner. Around 90% (n =409) were pregnant for the first time. At the time of interview, 87% reported that they were married, 12% were living together with their male partner and 1% were ‘single’.

**Table 1:** **Socio-demographic characteristics of the pregnant teenagers**

**Characteristic**	**Frequency**	**% distribution**
**Age in years**		
14	4	0.9
15	5	1.1
16	32	7.1
17	89	19.8
18	166	36.9
19	154	34.2
**Marital status at the time of interview**		
Married	392	87.1
Living together	53	11.8
Single, never married	5	1.1
**Ethnicity**		
Sinhalese	344	76.4
Tamil	54	12.0
Moor	52	11.6
**Religion**		
Buddhism	343	76.2
Hinduism	50	11.1
Islam	52	11.6
Catholicism/Christianity	5	1.1
**Educational level**		
No formal education	6	1.3
Grade 1-5	16	3.6
Grade 6-10	171	38.0
GCE(O/L) or equivalent	237	52.7
GCE (A/L) or equivalent	20	4.4
**Family income at the time of meeting partner**		
Deficit	68	15.1
Break even	198	44.0
Surplus, able to save	184	40.9
**Total**	**450**	**100**

The 150 male respondents comprised 37 partners of the pregnant teenagers in the study and 113 partners of other pregnant teenagers who were not interviewed for this study. Their ages ranged from 18–37 years (mean 24 years, SD 3.7), with 50% being 20–24 years and 9% over 30 years old. 57% of the partners had been educated to above grade 10 and 90% were fully literate. Just five (3%) could not read at all. The majority (84%) were employed, with 45% reporting semi-skilled occupations.

### Family setting

The vast majority of the girls had grown up in a home with married parents and reported that they were living with their mother (93%) and father (95%) at the time they met their current partner/husband. Over three quarters of the teenage respondents said they were ‘very happy’ and just 6% that they were ‘not happy’ as a teen prior to meeting the current partner. The majority reported good family relationships with 88% describing their relationship with their mother as ‘very close’ and 82% stating that it was ‘very easy’ to discuss matters of importance with their mother. Notwithstanding the general picture of stable and supportive family environments, comparisons between teenagers aged less than 18 years and those aged 18 or older showed a significant difference in the proportion reporting that they were ‘very happy’ in their teenage years (69% versus 80%, p = 0.011chi squared test) and also in those reporting that they found it ‘very easy’ to discuss matters with their mother (74% versus 86% p = 0.009 chi squared test).

### Sexual activity and relationship status

Among first time pregnant teenagers, the reported age of first sexual experience ranged from 13–19 years (mean age 16.6, SD 1.2). Fifteen percent reported first sexual experience (kissing, cuddling, petting etc.) when aged less than 16 years with 7% reporting first intercourse below 16 years (the age of consent in Sri Lanka).

Overall, the picture is predominantly one of young women who were willing participants in these sexual encounters. Over 90% said they had wanted the first intercourse to happen. Just ten respondents reported not wanting their first sexual intercourse and a further five that it had occurred forcefully. However, 69% of the total respondents and 93% of those under 18 years considered that they had been ‘too young’ at first intercourse, indicating some ambivalence towards early sexual activity.

Among the male partners, the reported age of first sexual experience ranged from 12–37 years (mean 21, SD 3.9) with age of first intercourse ranging from 16–37 years (mean 23, SD =3.7). Among those who reported that their first intercourse had been with the opposite sex (93%), they all reported that this had involved a girlfriend or wife. Furthermore, 87% of the men reported having had only one sexual partner, suggesting that the teenage pregnancies had occurred within monogamous and stable relationships.

The most common reasons given by pregnant teenagers for first intercourse were: being already married at the time (56%) and living with their partner or intending to get married (27%). These findings suggest that many of these first sexual encounters were with men who later became stable partners. Nevertheless, it is worth noting that reports from both the teenage sample and the partner sample indicated that in over 90% of cases male partners were older than the pregnant teenagers, and the age difference was substantial in a large proportion of cases, particularly for those girls in the younger age groups (Table 2).

**Table 2:** **Percentage distribution of age-gap between partners by age of pregnant teenager (N = 409)**

Age gap between partners (years)*	**Pregnant teenager’s age in years**						
	**14**	**15**	**16**	**17**	**18**	**19**	**Total**
Same age	0	0	0.0	6.0	7.2	12.5	8.1
< 3	0	20	31.1	26.4	30.9	30.9	29.6
3 to 5	0	0	10.3	13.3	10.6	11.8	11.2
6 to 9	25	60	41.3	38.6	36.2	33.8	36.4
10+	75	20	17.3	15.7	15.1	11.0	14.7
Total (N)	4	5	29	83	152	136	409

*Male age minus female age; teenagers’ reports.

### Planning and responses to first teenage pregnancy

**Figure 1:**
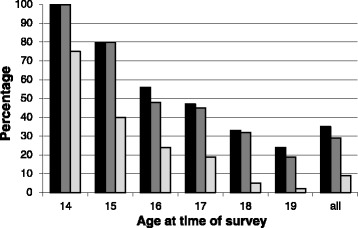
**Responses to first pregnancy by age of the respondent at the time of survey (pregnant teenagers, N = 409)** ***.*** Black - % reporting pregnancy as unplanned. Grey - % reporting ‘uncertain’ or ‘not pleased’ at news of pregnancy. Light grey - % reporting feeling ‘not happy at all’ since discovering pregnancy.

Figure 1 plots three variables side-by-side for the pregnant teenagers: the percentage of respondents who reported that their pregnancy was unintended/unplanned (including the 34 respondents who reported their pregnancy as ‘neither planned nor unplanned’); the percentage who reported that they were uncertain or ‘not pleased’ by the news of pregnancy; and the percentage who reported that since being pregnant they were feeling ‘not happy at all’, for the sample overall and according to the age of the respondent.

Overall, 64% of respondents stated that their current pregnancy was planned and intentional, with 8% reporting that the pregnancy was ‘neither planned nor unplanned’ and 28% stating that it was unplanned. However, there was a strong association with age, with 100% of respondents aged 14 and 80% of those aged 15 at the time of the survey reporting that their pregnancy was not planned compared to around 25% for 19 year-olds. Consistent with answers to the question on planning the pregnancy, around 60% of respondents stated they had not been using contraception because they intended to conceive, while only 23% had been using a method of contraception when they became pregnant (13% the oral pill, 9% natural methods, 1% condom).

Although only 64% of the teenagers had planned to become pregnant, 79% of the sample reported that they were ‘very happy’ since being pregnant, suggesting that for a majority of the adolescents pregnancy was not seen as a problem and that even for those that had not planned their pregnancy, most had subsequently adjusted to their situation.

A similar picture emerged from the male partner data, with around two-thirds reporting that the current pregnancy had been planned and just 29% indicating that pregnancy had occurred as a result of contraceptive failure. Again, rather more respondents - 85% - stated that they had welcomed the pregnancy; indicating that most had come to terms with the situation.

The majority of the pregnancies were proceeding in a supportive environment. Overall, 94% of the pregnant teenagers felt ‘very well supported’. The primary source of support most commonly reported by the pregnant teenagers was their partner/husband (95%). Many also mentioned their mother (90%) as a source of support as well as their father (78%). Responses from the male partners also suggested good support with 99% reporting receiving support from family and providing support themselves to their pregnant partner.

It is important to highlight, however, that the findings paint a different picture for the young girls in the sample. Among the 14 year olds, 75% reported being ‘not happy at all’ and for the 15 year olds this figure was 40%. It was also found that 26 (6%) respondents (across all the ages) who were in their first pregnancy wished to get rid of the pregnancy and 10 (2%) reported actually trying to do so. Similarly, it is important to note that seven respondents felt that they were not supported at all since becoming pregnant and all of these were younger than 18 years. Furthermore, the proportion of pregnant teenagers citing their parents as a source of support was found to be significantly lower among pregnant teenagers aged under 18 years than those aged 18 years plus (83% citing mother versus 93% and 67% citing father versus 82%, both p < 0.001 chi squared test). Similarly, among those reporting that their pregnancy was not planned, 16% reported feeling ‘somewhat supported’ or ‘not supported at all’, whereas 100% of those who had planned the pregnancy felt ‘very well supported’.

### Levels and sources of reproductive health knowledge

Reproductive health knowledge of the respondents was limited. Although 90% of respondents reported that it was possible for a girl to get pregnant after a sexual relationship with a man, only 46% recognised this risk at first unprotected sexual intercourse. Only 17% of the girls demonstrated satisfactory knowledge of the fertility period by correctly identifying when during the monthly menstrual cycle pregnancy is most likely to occur.

The male partners had reproductive knowledge levels that were better than those of the pregnant teenagers, with 64% aware of the possibility of pregnancy at first intercourse and 21% aware of the fertile period.

In terms of knowledge about contraception, respondents were asked, for each method in turn: whether they had heard of the method; knew how to use it; where to obtain it; and whether they could obtain it. Satisfactory knowledge was judged as positive answers to all four of these. The majority of the pregnant teenagers (86%) demonstrated satisfactory knowledge of at least one method. However, it is important to note that these answers are unlikely to reflect the respondents’ level of knowledge at the time they conceived since all the respondents would have been exposed to information on contraception during their interactions with healthcare professionals since their enrolment in antenatal care. Just over half (51%) reported that they had ever used contraception, with just 14% reporting use at first intercourse. Among male partners, contraceptive knowledge levels were slightly lower, with 80% having satisfactory knowledge of at least one method. The best known methods of contraception among both respondent groups were condoms and the pill. Just 15% of male partners reported that they had used contraception at their first intercourse. Knowledge of long-acting contraceptive methods was very low.

Among the pregnant teenagers, the most commonly cited sources of knowledge about puberty were: mother (61%) and school (41%). Only 13% identified friends. Mothers were also most commonly identified as the person with whom they could discuss problems related to puberty (with 80% of respondents mentioning their mother). Only 11% of respondents reported that they had discussed sexual relationships with friends and 12% that they had discussed how girls become pregnant with friends.

### Factors associated with unplanned teenage pregnancy

The analysis above suggests that a majority of teenage pregnancies in this context were welcomed. Nevertheless, a proportion of pregnancies were unplanned. The planned status of a pregnancy might impact upon the experience of pregnancy, its outcome and the subsequent wellbeing of mother and child. It was therefore of interest to explore the predictors of unplanned pregnancies.

A dichotomous variable was constructed placing those who reported their pregnancy as ‘planned’ (n = 263, 64%) in one category and grouping those who answered ‘unplanned’ with those who said ‘neither planned nor unplanned’ together into the other category (n = 146, 36%), henceforth referred to as ‘not planned’.

Variables explored in the regression analysis related to potentially explanatory pathways: socio-economic circumstances; family setting (relationship with and communication with mother); experiences of school and educational attainment (literacy; opinion of schooling; school performance); reproductive health knowledge and experience (knowledge of contraception; age at menarche; attitudes and experience of first sexual experience and intercourse); partner characteristics (partner’s age and age-gap between respondent and partner) and community norms (ethnicity).

Bivariate analyses using chi squared tests showed that the following factors were significantly associated with the pregnancy being reported as not planned: younger age; ethnicity (with pregnancies being more likely to be unplanned among Buddhist Sinhalese (38%) and Hindu Tamil (37%) than among Muslim Moor respondents (15%) p < 0.01); attitude to first sexual intercourse (with pregnancies being more likely to be unplanned among those reporting uncertain/negative attitudes (63%) than those who had wanted first intercourse (33%) p < 0.01); happiness as a teen (with pregnancies being more likely to be unplanned among those reporting being unhappy/somewhat happy as a teen (52%) than among those reporting being ‘very happy’ (31%), p < 0.01); relationship with mother (with pregnancies being more likely to be unplanned among those reporting being ‘somewhat close/not close’ (50%) than among those reporting a ‘very close’ relationship (34%) p = 0.03); and communication with mother (with pregnancies being more likely to be unplanned among those reporting discussion as ‘somewhat easy/not easy at all’ (51%) than among those reporting discussion as ‘very easy’ (32%) p < 0.01).

Results of the logistic regression analysis conducted to identify factors associated with pregnancy being not planned are presented in Table 3. Variables were selected by looking at the unadjusted odds ratio and if the p value was less than 0.25 they were entered into the model. Ethnicity, economic status, literacy, opinion of schooling, school performance, adolescent’s relationship with the mother, discussion with the mother, happiness as a teen, attitude to first sexual intercourse, age at menarche, partner’s age and age gap between respondent and partner all had a p value of less than 0.25 and were introduced into the stepwise model.

**Table 3:** **Forward stepwise logistic regression model on factors related to ‘not planned’ pregnancy (first time pregnant teenagers N = 409)**

		**B**	**S.E.**	**p**	**OR**	**95.0% C.I. for OR**	
						**Lower**	**Upper**
Step 1^a^	Not happy as a teen	.868	.239	.000	2.38	1.49	3.81
	Constant	-.805	.122	.000	.45		
Step 2^b^	Intercourse not wanted	1.181	.401	.003	3.26	1.49	7.14
	Not happy as a teen	.841	.242	.001	2.32	1.44	3.76
	Constant	-.890	.127	.000	.41		
Step 3^c,d^	Ethnicity			*.009*			
	Tamil	-.222	.335	.508	.80	0.415	1.55
	Moor	−1.317	.434	*.002*	.27	0.11	0.63
	Intercourse not wanted	1.259	.415	*.002*	3.52	1.56	7.94
	Not happy as a teen	.820	.249	*.001*	2.27	1.39	3.69
	Constant	-.742	.136	.000	.48		

^a^Variable(s) entered in step 1: Teen happiness. Base line: Very happy as a teen.

^b^Variable(s) entered in step 2: Attitude to first intercourse. Base line: first intercourse wanted.

^c^Variable(s) entered in step 3: Ethnicity. Baseline: Sinhalese.

^d^Hosmer and Lemeshow goodness-of-fit test, p = 0.993. Nagelkerke’s R^2^ = 0.109.

Table 3 presents the step-wise model against a constant-only model showing a statistically significant difference. This indicates that the determinants as a set distinguished between those who planned their pregnancies and those who did not (chi square =33.838, p < .001) with df =4. The Wald criterion demonstrated that, adolescent’s ethnicity (p = .009), not wanting the first intercourse (p = .002) and not being happy as a teen (p = 0.001) were all statistically significantly associated with the pregnancy being not planned. Household economic status, literacy, perceived school performance, relationship with the mother, discussion with the mother, age at menarche, age of the partner and age difference between the partners were not found to be independently significantly associated with this outcome (p values all greater than 0.05). The goodness-of-fit test indicated that the model was an adequately fitting model, but the Nagelkerke’s R^2^ indicates that only 10% of the variation in outcome can be accounted for by these variables.

The odds ratios indicate that among this sample of pregnant teenagers, those who reported that they did not want their first intercourse to happen had odds of reporting their pregnancy as not planned three times higher than those who had wanted their first intercourse. Also, those who reported being less than ‘very happy’ as a teen had odds of reporting their pregnancy as not planned two times higher than those who had been ‘very happy’. Compared to the Sinhalese and Tamils, Moor adolescent girls were significantly less likely to report their pregnancy as not planned (OR 0.73; p < 0.05).

## Discussion

The findings presented above offer important new insight into the context in which teen pregnancy occurs in Sri Lanka incorporating perspectives of both pregnant adolescents and their male partners. Sri Lanka presents a very different socio-cultural context from Western settings where much prior research into teenage pregnancy has been conducted, with strong proscriptions against pre-marital sex and an exceptionally low incidence of sexual activity among school-going adolescents by international standards [25]. At the same time, Sri Lanka diverges in important ways from its South Asian neighbours, with much higher rates of school attendance, particularly for girls, and later female age at marriage and lower rates of adolescent pregnancy than in India, Bangladesh or Pakistan [15]. As such, extrapolation of evidence from other contexts is problematic, and this paper contributes importantly to our emergent understanding of adolescent pregnancy in the Sri Lankan setting.

The dominant picture is one where teenage pregnancies are either planned or, if not planned, welcomed, and where teenage pregnant girls are living within stable and supportive family environments. These findings support and extend other recent work that reports many teenage pregnancies to be planned [6] and most teenage mothers to have parental support [3]. Discrepancies between the proportions who reported they were married at the time of the survey (87%) and the proportion who had achieved the legal age of marriage of 18 years (71%) suggest some falsification of age. This situation reflects the important social respectability that marital status confers on these girls (as previously identified by Waidyaratne [26]) and indicates a socio-cultural context that accommodates teenage pregnancy. Recent qualitative work in other parts of the country also suggests parental complicity in under-age unions [13]. In this context, it could be argued that, for many of these pregnant adolescents who are likely to have grown up with some degree of poverty and have limited career prospects, delaying pregnancy would be unlikely to make a significant difference to their life trajectories whilst pregnancy offers them social status in a culture where motherhood remains core to a woman’s identity [27]. This appears to be particularly the case among the Muslim Moors, among whom few pregnancies were reported as unplanned. Clearly, the normative status of teenage pregnancy among these communities contrasts starkly to contexts where such pregnancies are heavily stigmatised, such as in the UK and US [28,29], though it should be remembered that even in Western settings teenage pregnancy is not considered problematic by all sections of the population [30]. Nevertheless, even in this setting where adverse outcomes of pregnancy are minimised by high quality maternity care that is accessed by almost all women [19], pregnancy at a very young age still raises concerns for maternal and infant health, as evidenced in several earlier studies [31,32]. Furthermore, it was important to note that a large proportion of respondents, while welcoming their pregnancy, nevertheless felt that they had engaged in sexual relationships at ‘too young’ an age, perhaps suggesting that while they are accepting of their current situation, they can nevertheless envisage an alternative life trajectory that involved delayed sexual activity and reproduction. This finding resonates with Fernando et al.’s finding that a majority of pregnant teenagers in their sample reported that the decision to have a baby had been taken by their husband [6].

Notwithstanding a generally positive picture, our findings do suggest a sub-group of pregnant teenagers who are vulnerable. Consistent with earlier hospital-based studies [3,5], a significant minority of girls were not happy in their pregnancy. Moreover, reporting an unplanned and unhappy pregnancy was strongly associated with first intercourse being reported as not wanted. The majority of these adolescents were younger than 18 years of age suggesting a clustering of vulnerability. The finding that some pregnant teenagers had tried to get rid of the pregnancy is a matter of concern as abortion is only legally sanctioned in Sri Lanka when the mother’s life is endangered. The marked age difference between girls and their partners was a notable finding. Both components of the study reported a situation where over 50% of the pregnant teenagers had a partner six or more years older than them. The younger the teenager, the more likely they were to have a partner considerably older than themselves, a factor that may contribute to the potential vulnerability of these young girls.

Both the girls and the male partners demonstrated low levels of reproductive and contraceptive knowledge, in spite of the fact that respondents would have had the opportunity to be exposed to information from healthcare professionals during their antenatal care. These findings, together with the fact that half the girls reported never using contraception, very few reported use at first intercourse and nearly one quarter reported contraceptive failure, indicate a group with insufficient knowledge and service access for informed reproductive decision making. Those respondents with unplanned and unwanted pregnancies – though a small group numerically – were disproportionately represented in the youngest age groups and may be particularly vulnerable, indicating the need for action to improve their knowledge of, access to and ability to use, contraception. The findings also suggest missed opportunities during the antenatal period to provide knowledge that could support the delay of the next pregnancy. This is confirmed by the substantial proportion of teenage girls in the sample (almost 10%) who were in their second pregnancy. Male partners also showed low levels of knowledge about contraception.

When sources of information and support are considered, the findings show that the mother of the pregnant teenager plays a key role; with friends and peers being mentioned much less often. This contrasts with findings from other settings, where the significance of peer networks and friends is often emphasised [33,34], and raises important questions regarding effective routes to increasing knowledge levels and access in this context.

There are a number of strengths and limitations of the study. On the positive side, the availability of a robust sampling frame and the 100% recruitment rate for pregnant teenagers ensured the representativeness of the findings to the study district. Further, while the accuracy of self-reporting in administered questionnaires is indeterminate, the use of experienced and well-trained interviewers plus careful design and administration of the data collection tool aimed to minimise the possibility of social acceptability bias in responses. It was necessary to delay recruitment of partners because they were subject to legal scrutiny at the time of the study. As a result, only 15% of the males were the partners of the girls in the study. Although this limited direct comparability between the two data sets, the inclusion of male partners alongside pregnant teenagers has not previously been achieved in Sri Lanka and these data provided important additional insight into the topic.

It is recognised that, despite the very high antenatal care coverage, a small number of teenage pregnancies would not have been recorded on the midwives’ registers, either because they were concealed and/or terminated, and therefore would fall outside our study. Provision was made for the study team to be notified of any adolescents who attended the hospital for delivery who had not previously been registered with a midwife. Four such notifications were made during the study period, all of them adolescents who had come from outside of the study area, suggesting that the numbers of non-registered adolescent pregnancies that progress to term is very low. Identification and investigation of such pregnancies would require a qualitative method.

The extent to which the findings can be extrapolated to other parts of the country deserves careful consideration. Exploration of socio-economic indicators indicates that Badulla district is comparable with other parts of the country reported in the Demographic and Health Survey [19]. Also, findings from a school-based questionnaire that formed part of the present project and are reported elsewhere, show findings that are comparable with recent national data [35] giving confidence that Badulla is not exceptional in any important respect. Nevertheless, recent analysis underscores the importance of acknowledging regional variations in levels and patterns of teenage pregnancy and the danger of extrapolation [2]. A further point worth mentioning is that our multivariate model was unable to explain a large amount of the variation in planned versus not planned pregnancies despite our inclusion of a wide range of variables, suggesting important unobserved factors. Furthermore, the results raise a number of questions that require more qualitative methods of investigation, as well as a longitudinal design, including the implication of the pregnancy for the career and future socioeconomic wellbeing of the teenager and her male partner.

## Conclusion

The key messages for healthcare practice from this study relate to the importance of ensuring that pregnant teenagers and their male partners are provided with adequate contraceptive information and service access to ensure that they are able to delay their second pregnancy. Community midwives already provide care to the vast majority of pregnant teenagers and these contacts should incorporate more effective education for these girls and their wider families. In addition, there is a need to develop strategies for reaching out to those girls who are particularly vulnerable to unwanted sexual activity and pregnancy at a very young age.

In terms of policy development, the key message from this study is that efforts to reduce teenage pregnancy must recognise the normative nature of early childbearing for the majority of girls who currently conceive and the fact that parents and the wider community are commonly supportive and even complicit in arranging marriages below the legal age. Avoiding such pregnancies is not simply a question of providing education; it requires a more fundamental shift in life chances such that delaying pregnancy offers significant socioeconomic advantages for these young women and their families.

Finally, in terms of the research agenda, this study has highlighted the dangers of extrapolating learning from Western settings and suggests the need for conceptual and qualitative empirical work, to better characterise the context of teenage pregnancy in Sri Lanka and particularly to understand the factors that place some young women in particularly vulnerable situations.
